# The use of retroviral vectors for gene therapy-what are the risks? A review of retroviral pathogenesis and its relevance to retroviral vector-mediated gene delivery

**DOI:** 10.1186/1479-0556-2-9

**Published:** 2004-08-13

**Authors:** Donald S Anson

**Affiliations:** 1 Department of Genetic Medicine, Women's and Children's Hospital, 4^th ^Floor Rogerson Building, 72 King William Road, North Adelaide, South Australia, 5006, Australia; 2 Department of Paediatrics, University of Adelaide, South Australia, 5005, Australia; 3 Department of Biotechnology, Flinders University, GPO Box 2100, Adelaide, South Australia, 5001, Australia

## Abstract

Retroviral vector-mediated gene transfer has been central to the development of gene therapy. Retroviruses have several distinct advantages over other vectors, especially when permanent gene transfer is the preferred outcome. The most important advantage that retroviral vectors offer is their ability to transform their single stranded RNA genome into a double stranded DNA molecule that stably integrates into the target cell genome. This means that retroviral vectors can be used to permanently modify the host cell nuclear genome. Recently, retroviral vector-mediated gene transfer, as well as the broader gene therapy field, has been re-invigorated with the development of a new class of retroviral vectors which are derived from lentiviruses. These have the unique ability amongst retroviruses of being able to infect non-cycling cells. Vectors derived from lentiviruses have provided a quantum leap in technology and seemingly offer the means to achieve significant levels of gene transfer *in vivo*.

The ability of retroviruses to integrate into the host cell chromosome also raises the possibility of insertional mutagenesis and oncogene activation. Both these phenomena are well known in the interactions of certain types of wild-type retroviruses with their hosts. However, until recently they had not been observed in replication defective retroviral vector-mediated gene transfer, either in animal models or in clinical trials. This has meant the potential disadvantages of retroviral mediated gene therapy have, until recently, been seen as largely, if not entirely, hypothetical. The recent clinical trial of γc mediated gene therapy for X-linked severe combined immunodeficiency (X-SCID) has proven the potential of retroviral mediated gene transfer for the treatment of inherited metabolic disease. However, it has also illustrated the potential dangers involved, with 2 out of 10 patients developing T cell leukemia as a consequence of the treatment. A considered review of retroviral induced pathogenesis suggests these events were qualitatively, if not quantitatively, predictable. In addition, it is clear that the probability of such events can be greatly reduced by relatively simple vector modifications, such as the use of self-inactivating vectors and vectors derived from non-oncogenic retroviruses. However, these approaches remain to be fully developed and validated. This review also suggests that, in all likelihood, there are no other major retroviral pathogenetic mechanisms that are of general relevance to replication defective retroviral vectors. These are important conclusions as they suggest that, by careful design and engineering of retroviral vectors, we can continue to use this gene transfer technology with confidence.

## Background

### Retroviruses

Retroviruses are viruses that are found throughout the animal kingdom, including in chickens, mice, cats, sheep, goats, cattle, primates, fish and humans. The first retro viruses were identified as cell free oncogenic factors in chickens. Subsequently, many of the oncogenic retroviruses have been shown to be replication defective forms that have substituted a part of their normal viral gene complement with an oncogene sequence [1]. Replication competent retroviruses also cause malignant disease, as well as a range of other pathogenic states, in a broad range of species. This includes what must be the most significant transmissible disease of humans in recent times, acquired immunodeficiency syndrome (AIDS), which is caused by the retroviruses Human Immunodeficiency Virus Types 1 and 2 (HIV-1, HIV-2). However, many retroviruses cause life-long infections and appear to be relatively, if not completely benign, in their normal host species. In mice there are retroviruses that are very closely related to strongly oncogenic retroviruses but which are not themselves oncogenic, or are only very weakly oncogenic [2-5]. In addition, there is a whole class of retroviruses, the spumaviruses, or foamy viruses, which do not appear to be linked to any specific pathogenic state [6]. Even the simian equivalent of HIV-1, the causative agent of AIDS, is not pathogenic in all its hosts [7]. There is also a range of endogenous retroviral sequences that are not associated with specific pathologies [8]. Vestigial forms of retroviruses also exist; these are represented by various classes of insertional elements and can constitute a significant proportion of animal genomes [8].

The retroviral virion is a spherical particle of about 80–100 nm in diameter. It is enclosed by a lipid bilayer derived from the host cell plasma membrane into which one of the retroviral gene products, the envelope protein, is inserted. The virion has considerable internal structure that is mainly comprised of the products of the viral *gag *gene. In addition, the virion contains two *identical *copies of a genomic RNA molecule (the retrovirus is then genetically haploid but can also be described as pseudo-diploid), a tRNA primer for reverse transcription as well as small amounts of the products of the viral *pol *gene. The virion may also include a range of other host cell derived proteins although it is unclear whether these represent a random assortment of proteins that are coincidently incorporated into the virion or whether they play some role in the viral life cycle. Both possibilities are probably true, certainly HIV-1 is known to incorporate into its virion a number of host cell proteins that play a vital role in its life cycle [9,10].

While the simple retroviruses have only three genes, *gag*, *pol *and *env*, the complex retroviruses encode a number of other proteins that are involved in regulating viral replication or the host cells response to the virus. For example, HIV-1 has six gene sequences in addition to the minimal retroviral complement of *gag*, *pol *and *env*. Two of these, *tat *and *rev*, encode proteins that regulate expression of the viral genome, while the other four, *vpu*, *vif*, *vpr *and *nef*, encode proteins that play multiple roles in enhancing viral replication.

### Retroviral life cycle

It is the unique nature of the retroviral life cycle, combined with the simplicity and advantageous arrangement of the retroviral genome, which has made retroviruses so attractive as vectors for gene therapy [11,12]. The principal feature of the retroviral life cycle that is of interest is the ability of the retrovirus to copy its RNA genome into a double-stranded DNA form which is then efficiently and exactly integrated into the host cell genome. The integrated form is termed the provirus and it is transcribed as a normal cellular gene to produce both mRNAs encoding the various viral proteins, and the genomic RNA that is packaged into progeny virions.

**Figure 1:**
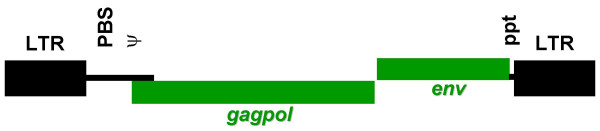
**The *cis *and *trans *genetic functions of a retrovirus. ***Cis *sequences (shown in black) are those that are directly active as nucleic acids, they include the 5' long terminal repeat (LTR) which, in the DNA form found in the provirus acts as a transcriptional promoter, and in the RNA (genomic) form contains sequences important for reverse transcription of the genome; the primer binding site (PBS) for first strand DNA synthesis during reverse transcription; the psi (ψ) sequence which directs packaging of the genomic RNA into the virion; the polypurine tract (ppt) which is the primer binding site for second strand DNA synthesis during reverse transcription and the 3' LTR which, in the DNA form (in the provirus) acts as a polyadenylation signal, and in the RNA (genomic) form contains sequences important for the reverse transcription process. The *trans *functions (shown in green) are the protein coding sequences, these are the *gagpol *gene, which encodes the Gag and Pol polyproteins, and the *env *gene that encodes the viral envelope protein.

**Figure 2:**
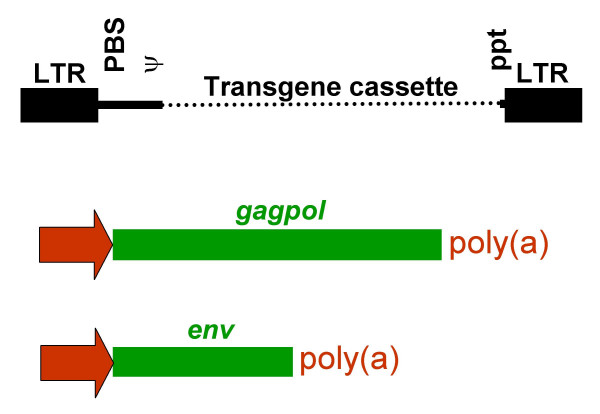
**Separation of the *cis *and *trans *functions of a retrovirus in a recombinant, replication defective vector system. **Replication defective retroviral vector systems are made by separating the *cis *(shown in black) and *trans *(shown in green) genetic functions of the virus into a vector construct, which contains the *cis *sequences, and helper or packaging plasmids, that encode the viral proteins (i.e. contain the *trans *sequences). To minimize overlap between the two components of the system heterologous transcriptional control elements (shown in red) are used to express the *trans *functions. Recombinant virus is made by introducing all these elements into the same cell. Only the vector transcript is incorporated into virions as this is the only RNA that contains the retroviral packaging signal (ψ).

The genetic structure of the virus and the existence of the proviral form make it easy to manipulate retroviruses to make replication defective vectors for transfer of heterologous gene sequences. The proviral form, being DNA, can be readily isolated in standard plasmid cloning vectors and so made amenable to molecular manipulation. The genetic structure of the virus is such that the viral *cis *(sequences that are biologically active in the form of nucleic acids) and *trans *(protein coding sequences) functions (Fig. 1) are largely non-overlapping; indeed, as far as recombinant vectors are concerned it is possible to separate them completely, albeit at some cost in efficiency. The generation of systems capable of producing non-replication competent virus can then be achieved by placing the *cis *elements on a transfer vector construct and expressing the *trans *functions using standard recombinant plasmid expression systems (Fig. 2). As the genomic RNA expressed from the transfer vector construct is the only RNA molecule that carries the *cis *signals required for packaging into the virion, and for reverse transcription and integration, no viral genes are transferred to cells infected with the resulting virus. The resulting provirus, lacking all viral genes, is a replicative dead end and no further viral replication is possible. The nature of the retroviral replication process, where the U3 region of both the 5' and 3' LTRs of the provirus are effectively copied from the 3' LTR of the provirus in the preceding generation, also makes possible the construction of self-inactivating (SIN) vectors. With these vectors the resulting provirus contains no active retroviral derived transcriptional promoter or enhancer elements [13,14].

The use of a replication-defective retroviral vector to transfer gene sequences into target cells has been termed *transduction*, to distinguish it from the process of *infection *with replication competent viruses. It is theoretically possible that with most, if not all, recombinant vector systems, that recombination of the constituent parts of the system with each other, or with cellular sequences, can regenerate a replication competent retrovirus (RCR) [15,16]. However, the careful engineering of these systems has led to the point where they can largely be assumed to be free of such RCR. While this does not mean that screening for RCR in preparations of vector is unimportant, as there are a number of other ways in which RCR may arise, and as quality control is obviously central to the clinical use of retroviral vectors, it does mean that in practice RCR generation should no longer be a major safety issue. This means that in terms of evaluating the safety of retroviral vectors it is the direct and indirect consequences of proviral integration that are important to consider, rather than the effects of actively replicating virus.

### Retroviral mediated pathogenesis

Retroviruses have historically been most intensively studied in animals that are either the subject of scientific experimentation (principally the laboratory mouse), or are of commercial significance (such as farmed animals such as chickens, horses, goats, cattle and fish, and pets), where they cause a number of commercially significant diseases. Indeed, the first retroviruses to be described were the oncogenic retroviruses Avian leukosis virus (ALV), and Rous sarcoma virus (RSV), which are both found in chickens. A large number of oncogenic retroviruses have now been described. These tend to cause malignant disease in a very high proportion of infected hosts. In addition, the complex retroviruses human T-cell leukemia virus (HTLV) and bovine leukemia virus (BLV) can cause leukemia in their hosts, although they do so in only a small percentage of infected individuals.

The lentiviruses are also overtly pathogenic and have been shown to be the causative agent of several slow progressive diseases in animals including arthritis and encephalitis in goats, leukemia in cattle, anaemia in horses, and immunodeficiency in cats, cattle, primates and humans. The AIDS epidemic means that the lentivirus HIV-1 is now the most intensively studied retrovirus ever-incredibly, given the relative genetic simplicity of the retroviruses, there appears to be much still to learn about many aspects of HIV-1. There are also a number of viruses that cause central nervous system (CNS) pathology. For some of these, such as HIV and HTLV, CNS disease is a secondary pathology, while others are more specific in their effects. Similarly, while ALV and RSV are best known as oncogenic viruses, they are also associated with wasting syndromes.

### Oncogenic retroviruses

The archetypal retroviral pathogen is the oncogenic retrovirus. Some of these are replication defective retroviruses that carry and express an oncogene sequence-indeed it was these retroviruses that largely allowed the concept of oncogenes to be first defined. These viruses induce cancers with relatively short latency periods. In addition, there are a large number of non-defective retroviruses that are oncogenic. These generally induce cancers after longer latency periods. HTLV and BLV and related viruses form a separate class of complex retroviruses that cause leukemia in a small percentage of infected individuals after very long latency periods. Retroviruses have also been associated with sarcomas in fish but these viruses have not been studied in great detail.

### Defective oncogenic retroviruses

These have been described in a number of species, but have been most extensively studied in the laboratory mouse. These are replication defective, simple retroviruses in which part of the normal viral genome has been replaced with a cDNA copy of a cellular oncogene. The viral oncogene sequence often contains mutations that make the protein it encodes act in a dominant manner. The capture of a cellular oncogene by a retrovirus is an extremely rare event, the major significance of these viruses in scientific terms is that they led to the discovery of cellular oncogenes. These viruses depend on the presence of a replication competent helper virus in order to replicate and they induce cancers with relatively short latency periods. The existence of a latency period suggests that oncogene expression is, in itself, not enough to cause malignant disease, but that additional genetic events are required. The majority of the cancers caused by these retroviruses are found in the haematopoietic system although sarcomas are also common. They are also able to transform the phenotype of cells grown in culture, principally by causing cells to lose their contact inhibition. The type of malignant event caused by any one virus is determined by the nature of the oncogene expressed by the virus and by the nature of the enhancer sequences present in the long terminal repeat which control the tissue specific expression of the oncogene.

Replication defective vectors obviously also have the same potential to capture oncogenes. However, the mechanism of oncogene capture by retroviruses, and its extreme rarity, means it is probably not of major relevance when considering the risk factors associated with the use of retroviral vectors for gene therapy.

### Non-defective oncogenic retroviruses

Non-defective, replication competent retroviruses are also associated with malignant diseases. These viruses do not carry oncogene sequences. Although first discovered in the chicken they have been most extensively studied in the laboratory mouse. These viruses induce cancer by activating cellular oncogenes *via *a number of different mechanisms. In contrast to the oncogene carrying retroviruses, these viruses are associated with much longer latency periods. This is a reflection of the relatively low probability that proviral insertion will result in activation of an oncogene, in combination with the requirement for other genetic changes before a cancer eventuates. Although proviral integration can also result in gene inactivation, inactivation of tumour suppressor genes does not appear to be a mechanism associated with any known instances of retroviral induced malignancy.

The principal routes of oncogene activation are transcriptional promotion from one of the viral LTRs, and activation of endogenous cellular promoters by the strong transcriptional enhancer elements present in the viral LTRs. In the former case the provirus must obviously integrate in the sense orientation and upstream of the relevant coding sequence. Transcription can be from either LTR [17], and may involve splicing from either the retroviral, or cryptic, splice donor sites to a splice acceptor within the gene sequence [17]. If transcription is from the 3' LTR it is usually associated with inactivating mutations in the 5' LTR [18]. Transcriptional enhancement can occur with the provirus in either orientation [19] and over relatively large distances [20,21]. This is by far the most common mechanism of oncogene activation. Another mechanism by which proviral integration can activate cellular oncogenes is by negation of negative regulatory elements in the oncogene or its transcript [22]. However, this is a rare phenomenon. If proviral integration is downstream of the oncogene translation initiation codon a dominant variant of the oncogene product may result [23].

Not all non-defective simple retroviruses are overtly oncogenic and the oncogenic, non-defective simple retroviruses show a spectrum of tissue specificity and oncogenic potential. Analysis of the oncogenic potential of different retroviruses has clearly shown that the major determinant of both the overall oncogenic potential of the virus, and the cell specificity of the type of cancer that results, is the viral long terminal repeat [24-27]. More specifically, it is the transcriptional enhancer sequences in the long terminal repeat that are the major determinant of these properties [28-33]. Mechanistically, this makes perfect sense. As transcriptional enhancer elements are capable of acting at a distance they will not only control transcription from the viral LTR but will also have the potential to influence transcription from promoter sequences in adjacent chromosomal genes.

In contrast to oncogene activation, the oncogenic potential of some retroviruses maps to the *env *gene sequences. For example, the SU protein (p55) of the polycythemic strain of Friend virus binds to, and activates, the erythropoietin receptor resulting in massive erythroid proliferation and splenomegaly [34]. However, p55 does not bind to the active site of the Epo receptor and the Epo receptor is not used as the receptor for virus infection. In fact, p55 is not a functional envelope for infection and a helper virus is needed to allow the virus encoding p55 to propagate itself. In an analogous manner, the *sag *gene of Murine Mammary Tumour Viruses (MMTV) induces an immune response by interacting with the T-cell receptor [35]. This does not result in leukemia but facilitates the eventual induction of malignant disease in an indirect way. As the interaction between Sag and the T-cell receptor is not *via *the antigen binding site itself, a large proportion of the T-cell population (up to 10%) is stimulated. This, in turn, stimulates B-cells, the initial cellular target for infecting MMTV, allowing enhanced viral replication and the subsequent infection of mammary epithelial cells, the eventual site of tumour formation. Although Sag is a major determinant of the oncogenic potential of MMTV it should be noted that in the final analysis malignancy is due to oncogene activation.

How HTLV [36] and BLV cause cancer is not entirely clear. Both are complex retroviruses, and in addition to the *gag*, *pol *and *env *genes common to all retroviruses, have two genes that encode regulatory proteins. HTLV causes adult T-cell leukemia, often after a very long latency period (two or three decades can pass between infection and emergence of malignant disease). Only a small percentage of infected individuals (about 1% for HTLV) develop cancer. Although the mechanism of disease induction is unclear it is certainly related to the clonal proliferation of infected cells *in vivo*. Although viral gene expression does not appear to be necessary for maintenance of the disease, evidence suggests that one of the regulatory proteins, Tax, is important in inducing the initial T cell proliferation.

Given the recent development of vectors from lentiviruses, including HIV, it is worth noting that despite intense scientific scrutiny, examples of insertional mutagenesis or gene activation resulting from infection with these viruses have not been documented. However, in the case of HIV-1 the limited lifespan of most infected cells means that this observation must be interpreted with caution.

In terms of replication defective retroviral vectors, the study of oncogenic retroviruses suggests that oncogene activation, *via *the provision of promoter or enhancer sequences, but especially the latter, will be the major risk factor for disease induction. In addition, selection of the retroviral envelope used for vector pseudotyping could also potentially play a role as could inadvertent transfer and expression of other retroviral proteins, at least for vectors developed from particular retroviruses, such as Friend virus.

### Retroviruses causing CNS disease

Several retroviruses cause CNS disease. Some of these, such as the murine retroviruses Cas-Br-E MLV [37] and FMCF98 [38] are specifically associated with CNS pathology. For other retroviruses, such as HTLV and HIV, CNS disease is not the defining pathology induced by the virus, even though for the latter a high proportion of infected individuals will develop CNS disease. Cas-Br-E MLV infects the brain *via *infection of the epithelial cells of the blood-brain barrier. After these become infected they release virus directly into the CNS where it infects microglial cells, resulting in a spongiform encephalopathy. The SU (env) protein has been shown to be a major determinant of the neuropathogenesis of Cas-Br-E MLV [39] and other neuropathogenic murine retroviruses. However, the mechanisms involved have not been elucidated although receptor activation [40], analogous to that caused by the SU protein of the polycythemic strain of Friend virus, has been suggested but as yet remains unproven.

HTLV causes CNS disease in only a small percentage (about 1%) of infected individuals after a latent period that can be as short as two, or as long as thirty years [41]. The development of CNS disease is not correlated with the development of ATL. For HTLV CNS disease is characterised by a vigorous inflammatory response involving T cells that causes severe demyelination in the spinal cord. Little is known about how the virus infects the CNS and what cell types are infected, or what factors influence the induction of CNS pathology.

Most individuals infected with HIV have virus within the CNS and the route of infection is thought to be transmigration of infected macrophages across the blood-brain barrier. As well as allowing opportunistic infections within the CNS there is a specific condition, AIDS dementia complex (ADC), which is a direct result of HIV infection of the CNS [42]. Within the CNS HIV is found in macrophages and microglia, and causes demyelination, vacuolation and gliosis. Again, the mechanism by which HIV causes CNS pathology is not well understood. The gp120 (Env) and Tat proteins have been shown to be neurotoxic *in vitro *and a number of the cytokines induced by HIV infection of monocytes and macrophages also have the capacity to damage neural tissue, either directly or indirectly [43].

All of the retroviruses that cause CNS disease would appear to do so as a consequence of their active replication. In the case of HIV there is direct evidence for this-treatment of patients with antiretrovirals can significantly decrease the severity of CNS disease [44]. However, aspects of CNS pathology remain unresolved, for example HIV encephalitis persists even during highly active anti-retroviral therapy [45]. Therefore, this area of retrovirus induced pathology does not appear to be of immediate relevance to replication defective retroviral vectors. However, until the mechanisms by which some aspects of CNS pathology are induced are better understood this facet of retroviral pathogenesis cannot be entirely dismissed in terms of its relevance to the design and use of retroviral vectors.

### Retroviruses causing immuno-deficiencies

The AIDS epidemic has brought a substantial focus to bear on the retroviruses that cause immunodeficiencies in general, and the subset of these that are lentiviruses in particular. Simple retroviruses that cause immune deficiencies in mice [46], cats [47] and primates [48,49] have been described. Somewhat surprisingly, the pathological mechanisms in these diseases are all different. In mice, immunodeficiency is associated with proliferation of B cells (the primary target of infection), macrophages and CD4+ T-cells, all of which are non-functional. The disease is consistent with the development of anergy after antigen driven stimulation of the immune response [50]. Expression of a mutant *gag *gene product, Pr60 Gag, which is not processed normally [51], is required for induction of disease. However, the pathogenetic mechanisms involved are not understood. The defect in Gag processing makes the virus replication defective and a helper virus is required for virus spread, although not for induction of disease [52].

In cats the simple retroviruses that induce immunodeficiency do so *via *expression of an altered SU (Env) protein. This protein is incapable of causing resistance to superinfection [53]; as a consequence repeated superinfection leads directly to T cell lysis [54] and immunodeficiency then results due to a loss of T-cell function.

The lentiviruses that have been associated with immune deficiency are FIV, SIV and HIV. All appear to share a common pathogenetic mechanism where virus infection of, and replication in, T-cells directly causes cell death, T-cell depletion and immunodeficiency [55]. Cell death is caused by high levels of viral replication in infected cells, although the exact mechanism is unclear. However, it is also clear that the pathogenesis of HIV-1 infection is much more complicated than this, with a complex interaction between the virus and host being played out over time [56]. In some non-human primates, infection with SIV is usually a chronic, but largely asymptomatic, condition [7]. This is thought to reflect a host/virus balance that has evolved over a long period of time. Presumably, the human AIDS epidemic reflects a recent movement of HIV into the population with a resulting imbalance between viral pathogenicity and host defences, which, after a relatively long period of infection, is resolved in favour of the pathogen.

Again, the pathogenetic mechanisms involved with these retroviruses do not have major relevance to replication defective retroviral vectors. However, the pathogenetic mechanisms involved in the murine and feline immunodeficiencies caused by simple retroviruses do reiterate the point that expression of certain retroviral gene products can induce serious pathogenetic states and that this fact may have some relevance to vector design.

### Lentiviruses

Apart from the lentiviruses mentioned above that result in immunodeficiency, there are a number of other lentiviral-associated diseases including those caused by caprine arthritis encephalitis virus (CAEV) [57], equine infectious anemia virus (EIAV) [58] and maedi/visna virus (MVV) [59]. For CAEV and MMV viral infection of macrophages seems to induce an inflammatory response involving macrophages and CD4+ and CD8+ T cells. It is this inflammatory response that is responsible for the different aspects of the pathology associated with infection by these viruses. EIAV causes erythrocyte lysis when high titres of cell free virus are present in the circulation. There are several mechanisms involved. Direct interaction of EIAV particles and erythrocytes results in complement mediated lysis and macrophage engulfment. This interaction is probably mediated by the Env protein. In addition, the virus appears able to suppress the differentiation of erythroid precursors. Eventually, most animals become asymptomatic carriers six to twelve months after infection.

For all these viruses pathology appears to be intimately linked to viral replication. Therefore, the pathological mechanisms involved are not of direct relevance to replication defective retroviral vectors.

### Other retrovirus induced pathologies

Retroviral infection has also been shown to be the cause of wasting and osteopetrosis in birds [60] and anaemia in cats [61]. Apart from feline anaemia, where the SU (Env) protein is a major, although not the sole determinant for the determination of pathology, the disease mechanisms involved are not well understood. However, pathology is clearly dependent on sustained viral replication meaning its significance to replication defective vectors is again limited.

### Pathogenic potential of retroviral vectors

From the known mechanisms of retroviral pathogenesis discussed above the most obvious pathogenic potential of retroviral vectors is (i) the production of a replication competent virus, and (ii) insertional mutagenesis, specifically oncogene activation. Clearly, the production of replication competent virus not only creates the potential of pathogenetic disease, but will also greatly increase the probability of insertional mutagenesis. In fact, in the one instance where a vector contaminated with a replication competent virus was administered to animals viral replication *per se *did not appear to have an overt pathogenetic affect, rather a T-cell lymphoma eventuated [62], presumably as a result of oncogene activation. Although these conclusions are obvious and widely acknowledged it is reassuring to know that there appear to be no retroviral pathogenetic mechanisms of general relevance to the safety, or otherwise, of retroviral vector systems that have been overlooked.

While the inadvertent transfer of *gag, env *and other retroviral genes also has the potential of inducing a pathogenetic state this would appear to depend on the specific retroviral gene sequence in question and to not be of general significance. Even so, minimizing the inadvertent transfer of retroviral gene sequences should clearly be an objective when developing retroviral vectors, not only because of this issue but also because it will have a bearing on the likelihood of replication competent virus being produced and of an endogenous retrovirus being activated.

In addition, even though oncogene capture by retroviruses is an extremely rare event, the very significant pathogenic potential of the viruses that result means that it should also be taken into consideration during the development of retroviral vector systems.

For various reasons, not least of which has been the problem of achieving positive experimental outcomes, only the issue of reducing the probability of replication competent virus arising has been systematically addressed during the development of retroviral vector technology. Indeed, great care has been taken in the development of retroviral vector systems to minimise the chance of producing replication competent retroviruses [63,64]. However, although clear means of doing so have been described [13,14,65], the need to minimize the probability of oncogene activation has often been made secondary to the issue of efficient transgene expression [66]. This has especially been the case with oncogenic retroviral vectors where transcriptional silencing has been a major problem [67].

### Replication competent virus

The generation of replication competent virus has, from the very beginning, been seen as the major safety issue for retroviral vectors and this has led to a prolonged effort to develop means of minimising the probability of it arising. There are two principal ways in which replication competent virus can be produced. The first of these is through recombination of the constituent parts of the vector system (i.e. vector and helper *trans *function plasmids), either with themselves or with endogenous viral sequences in the cell lines used for virus production [15,16]; the second is by activation of an endogenous proviral sequence. The first of these issues has been addressed by (i) breakdown of helper functions onto different plasmids; (ii) manipulation of codon usage in helper plasmids; (iii) removal, or mutagenesis, of unnecessary *cis *sequences present in the vector; (iv) the development of SIN vectors; (v) the minimisation of homology between the separate plasmids that make up the system; and (vi) the use of cell lines that do not contain endogenous retroviral sequences with homology to the vector system [13,14,63-65]. Although for many vector systems each of these approaches requires further refinement, in principle, they clearly provide the basis for the construction of vector systems where the probability of replication competent virus being produced *via *any of these mechanisms appears to be remote. While this doesn't negate the need for appropriate quality control procedures, especially as there is still the remote probability of inadvertent activation of an endogenous retrovirus from the cell line used for virus production, it means that the major safety issue faced by those wishing to use retroviral vectors is that of insertional mutagenesis and oncogene activation.

### Insertional mutagenesis and oncogene activation

As discussed above, oncogene activation can occur either by transcription from one of the proviral LTRs, or by activation of an endogenous promoter by provision of transcriptional enhancer elements. The transgene aside, these events would appear to depend absolutely on the presence of active transcriptional control elements in the viral LTRs as evidenced by the critical role LTR sequences play in determining the ability of most non-defective retroviruses to induce cancers, and in determining the tissue specificity of cancer induction. There is no evidence that retroviruses contain transcriptional control elements of significance in other parts of their genomes. Therefore, the main approaches to minimizing the probability of oncogene activation must be the development of vectors from non-oncogenic retroviruses, the careful development of the SIN vector principal, and careful consideration of the promoter used to drive transcription of the transgene (see below).

### Retroviral gene transfer

The minimization of the inadvertent transfer of retroviral genes to target cells is clearly a worthwhile objective as some of these genes have direct pathogenic potential and they may also influence the probability of endogenous retroviral sequences in the target cell being activated. Generally, the principles applied to the design of vector systems in order to minimize the probability of RCR being produced will also minimize the probability of inadvertent retroviral gene transfer. However, as the production of RCR requires multiple recombination events more effort should be made to analyse the rate of transfer and expression of individual retroviral gene sequences by vector systems. It is clear that the rate of individual gene transfer is much higher than the rate of RCR generation and can occur at a significant frequency even in highly evolved systems where RCR cannot be detected [68]. This suggests that further efforts need to be made to assess and reduce the rate of transfer of retroviral genes.

### Oncogene capture

The mechanism of oncogene capture appears to be dependent on the generation of a chimeric retroviral-oncogene transcript (69, 70). This suggests that the risk of oncogene capture will be related to the efficiency of termination/polyadenylation of the proviral transcript and that this should be considered and assessed in the process of vector development, especially as retroviral polyadenylation sequences are often relatively inefficient, perhaps reflecting the necessity for the polyadenylation signal to be inactive in the context of the 5' LTR. However, in transient virus production systems, where the transfected vector plasmid presumably remains either entirely, or almost entirely extrachromosomal, this mechanism would appear to preclude the probability of oncogene capture. In the case of stable producer cell lines there is clearly an argument for categorizing the integration site of the vector sequence and discarding any clones where this is in a known or suspected oncogene.

### Adverse events in animal experiments and clinical trials

The adverse events that have been observed in animal experiments and clinical trials reinforce the conclusions discussed above, that replication competent virus [62] and insertional mutagenesis [71,72] are the two risk factors of significance in retroviral mediated gene therapy. The two known instances where insertional mutagenesis/oncogene activation has resulted from the administration of a replication defective retroviral vector suggest that, the design of the vector aside, there are additional risk factors that influence the probability of an adverse event, the most obvious of these being the specific transgene expressed from the vector [71,73,74] which in both cases is a gene capable of influencing cell growth (although in neither case can it be considered a classical oncogene). In terms of the influence of vector design it is interesting to note that in both of these instances the same vector, pMFG [66] was used [75,76]. This vector is derived from MoMLV, a strongly oncogenic murine retrovirus, and notably uses the viral LTR to drive expression of the transgene. In both cases the vector appears to have been chosen primarily for its ability to efficiently drive transgene expression in haematopoietic lineages without consideration that this may also select for an increased risk of oncogene activation. Given the historical difficulty of obtaining good transgene expression from MoMLV derived vectors in haematopoietic lineages, and the lack of evidence to suggesting that oncogene activation was a significant safety issue with replication defective MoMLV vectors, it is not surprising that this approach was taken. Indeed, it is generally believed that, in general, the risk of insertional mutagenesis, while poorly defined, is probably substantially lower than seen in the X-SCID trial [77] where there appear to be a number of specific secondary risk factors [72-74]. In the absence of such secondary risk factors it is unclear what the real risk is; given the complexity of cellular and genetic regulatory processes and networks it is also unclear how many apparently innocuous transgenes will in fact increase the risk of adverse effects when expressed in a constitutive manner. However, no adverse events have been reported for the long running ADA-SCID trial where mature T-cells were targeted [78] or in PBL and PHSC targeted gene therapy for the same condition [79], although in both cases the number of patients who have been treated is very small. In all these protocols a non-self inactivating MoMLV derived vector was used. However, even with these unknowns it is apparent that improvements in vector technology, such as the use of SIN vectors, will greatly reduce the risk, whether or not additional risk factors are present.

In terms of the vector technology used on the two occasions where oncogene activation has been observed the following comments can be made:

1) The vector is derived from MoMLV and uses the LTR sequence to drive the transgene *via *splicing. MoMLV is a strongly oncogenic, non-defective virus that causes B-and T-cell lymphomas and leukemias in mice. As with other non-defective oncogenic retroviruses the primary determinant of its pathological properties is the long terminal repeat enhancer. MoMLV has been shown to induce oncogenesis *via *activation of any one of a number of different cellular genes (*Ahi1*, *Bla1*, *Bmi1*, Cyclin D2, *Dsi1*, *Emi1*, *Ets1*, *Evi1*, *Gfi1*, c-Ha-ras, *Lck*, *Mis2*, *Mlvi2*, *3 *and *4*, c-*myb*, c-*myc*, N-myc, *Notch1*, *Pal1*, *Pim1 *and *2*, prolactin receptor, *Pvt1*, *Tiam1 *and *Tpl2*).

2) The vector LTR is used to control transcription of the transgene. In the case of the X-SCID trial there is a strong selective pressure for gene corrected cells and accordingly there will clearly be an equally strong selection for transduced T-cell clones in which the LTR is active.

3) The PHSC is notoriously difficult to transduce with oncogenic retroviral vectors and the protocol used was designed to enhance transduction by using multiple cytokines to stimulate division of PHSC. This is likely to induce many genes involved in regulating cell growth. As retroviruses preferentially integrate into active gene sequences, this would increase the number of growth regulating genes accessible as targets for provirus integration and hence promiscuous, unregulated activation. Specifically, LMO2, the oncogene activated in the X-SCID trial, is normally expressed in primitive haematopoietic cells (the target for gene transfer) but not in mature cells (80). Therefore, it will be accessible for proviral integration during the transduction process and its continuing expression in maturing T cells generated from gene corrected precursors is biologically inappropriate.

The problems that occurred in this X-SCID trial, their broader relevance and possible answers, have all been reviewed from a number of aspects [72,73,77]. However, the focus has been on the biology of the system, and little attention has been paid to how technological changes in vector delivery systems and protocols might impact on the risk of insertional mutagenesis/oncogene activation.

Given what is known about retroviral mediated insertional mutagenesis it is surprising that more attention has not been paid to the technology used in many of the retroviral mediated gene therapy animal studies and human trials. With hindsight, it seems that the technologies used were selected on the basis of efficacy, not safety, that is achieving adequate gene expression took preference over consideration and assessment of insertional mutagenesis. However, given the technical difficulties involved in developing a workable protocol this is not surprising, and it is a pre-occupation that was, and is, shared by all gene therapy researchers.

Possible technological approaches that would appear to provide answers to these issues include:

1) The use of self-inactivating (SIN) vectors would make a major difference in that the provirus would lack all U3 enhancer sequences, negating the ability of the LTR to activate cellular genes. The vector should also not contain active splice signals. However, given the ability of SIN vectors to be repaired at a significant rate during virus production (see below) careful selection of the retrovirus used to build the vector backbone is also important if this risk is to be minimised. Clearly the construction of vectors from non-oncogenic retroviruses and the development of more effective (i.e. less prone to LTR repair) SIN vectors is warranted. If SIN vectors are to be used the transgene must be expressed from an internal promoter which must also be presumed to have the potential for oncogene activation. Therefore it would be preferable to use a promoter without highly active enhancer elements. In addition, the wisdom of incorporating matrix/scaffold attachment regions into vectors to increase expression may also be contraindicated as these sequences have long-range enhancer like properties (81). If high levels of gene product are required, consideration should be given to other means to enhance transgene expression, such as codon-optimisation of coding sequences.

2) Vectors should be developed from non-oncogenic retroviruses. The recent development of vectors from HIV-1 and other lentiviruses for unrelated reasons (predominantly their ability to transduce non-cycling cells) means that this has already happened. The Tat dependence of the HIV-1 LTR may also provide an extra measure of safety as long as Tat is not transferred along with the vector. However, the enhancing properties of the HIV-1 LTR in the presence and absence of Tat needs to be carefully defined in order to test the assumption that the HIV-1 LTR lacks the ability to *trans*-activate adjacent promoters. The different integration specificities of lentiviral (centrally in active gene sequences) and oncogenic (promoter adjacent in active gene sequences) retroviruses and vectors [82] also give reason to suppose that the former may be less likely to cause oncogene activation. However, this remains to be directly demonstrated.

3) The incorporation of strong transcription termination/polyadenylation signals and gene isolator sequences (83) may provide another means to reduce the possibility of adjacent genes being activated. These sequences should also reduce the probability of oncogene capture in virus producer cells. However, the incorporation of insulator sequences appears to lead to a significant loss of vector titre (84).

4) When the transgene plays a role in regulating cell growth, extra consideration should be given to using the relevant control signals from the gene in question to regulate expression of the transgene.

5) Although the PHSC is theoretically a very attractive target for gene transfer it is extremely difficult to transduce with retroviral vectors derived from oncogenic viruses such as MoMLV. Although efficient transduction of human PHSC can now be achieved this requires exposure to multiple cytokines over a relatively long culture period. The potential of new retroviral vectors derived from lentiviruses (85) and spumaviruses (86) to transduce PHSC with shorter exposure to less cytokines needs to be fully explored.

6) In general the limitations of vectors should be taken into account when designing gene therapy protocols. For example, in the case of X-SCID, it may be just as efficacious to target a more committed T-cell precursor that can be transduced more easily, and without biological manipulation using multiple cytokines. Alternatively, if the PHSC is to be targeted as highly enriched a PHSC population as possible should be used in order to expose the patient to the minimum number of transduction events compatible with the desired outcome. In the two X-SCID patients who developed T cell leukemia, molecular analysis of samples collected before the appearance of malignant disease showed the presence of >50 γc transduced T cell clones. Approximately 14 to 20 million transduced CD34^+ ^cells were infused into these patients. Therefore, it would appear the patient is exposed to a much greater number of transduced cells than is theoretically necessary to produce the desired result. In other words, the process of generating gene corrected T cell clones by transduction of CD34^+ ^cells is very inefficient.

### SIN vectors, how good are they?

With hindsight SIN vectors [13,87,88] now appear likely to be one of the most important general developments in retroviral vector technology since the advent of replication defective vector systems in the 1980s. SIN vectors take advantage of the reverse transcription reaction in which the U3 region of the 3' LTR acts as the template for the U3 region in *both *LTRs of the provirus. As the transcriptional enhancer elements in the 3' LTR are redundant in the context of a retroviral vector construct they can theoretically be deleted without affecting vector performance. After transduction of the target cell both LTRs are deleted and are transcriptionally silent. Although this requires that an internal promoter is used to control expression of the transgene, and makes it more difficult to generate high titre stable packaging cell lines, the advantages of the approach are obvious. However, SIN vectors have not been widely used in the case of oncogenic retroviral vectors, principally because viral titres were low, because of high rates of repair of the SIN deletion [13,89] and because of negative effects of the SIN deletion on gene transfer efficiency [90]. Subsequently, by the use of a heterologous promoter in the 5' LTR an effective SIN vector based on spleen necrosis virus was developed [91] but this vector has not been widely utilized to date.

In contrast, SIN vectors have been widely adopted in the lentiviral vector field [14,65,92,93] where transient expression systems are generally used to produce virus, avoiding the difficulties of making stable cell lines associated with SIN vectors. In addition, in terms of transgene expression, lentiviral SIN vectors appear to perform as well as, if not better than, vectors with an intact 3' LTR [93,94]. However, even with vectors with large 3' LTR deletions it is obvious that repair of the SIN deletion also occurs at a significant rate with lentiviral SIN vectors [14,92]. Therefore, while the concept of SIN vectors is a powerful one, further development and rigorous testing of this technology is required before it can be confidently used to address the problems of insertional mutagenesis.

## Conclusion

The most important determinant of the safety of retroviral vectors remains ensuring they are free of replication competent retrovirus of any sort. Clearly, the technologies available for the production of vector virions would appear able to preclude the production of replication competent virus by recombination of the constituent parts of the vector system (i.e. vector and helper plasmids) with a very high degree of certainty. However, production of replication competent virus from the cell lines used for virus production remains a theoretical possibility and more work needs to be done on generic assays for replication competent retroviruses.

Apart from the issue of replication competent virus, analysis of the pathologies associated with retroviruses, and the results of the X-SCID trial, demonstrate that careful attention must be paid to the ability of sequences in retroviral vectors to activate transcription of genes adjacent to proviral integration sites. Although the use of SIN vectors will greatly reduce the risk of such events, given the predilection of current SIN vectors to be repaired during virus production these vectors need to be further developed, especially for vectors derived from strongly oncogenic viruses. In addition, inadvertent transfer to, and expression in, transduced cells of *gag, env *(SU) and other retroviral gene sequences would appear to of relevance and needs to be specifically addressed in the development of vector systems.

As both oncogenic (MoMLV derived) and lentiviral (HIV-1 derived) vectors have been shown to preferentially integrate into transcribed sequences it would appear logical that the likelihood of proviral integration near cellular genes involved in the positive regulation of cell growth would be increased in actively growing cell populations. This suggests that the use of transduction protocols that target non-cycling cells, or cells that are subjected to the minimum of stimulatory signals as is compatible with efficient gene transfer, would be greatly advantageous in terms of minimising the risk of malignant events after the stimulatory signals are removed.

With hindsight, the observation of malignant events induced by replication defective MoMLV retroviral vectors is not surprising although the frequency of these events in the X-SCID gene therapy trail certainly was. The concern is that these events will now cause a significant backlash against the use of all retroviral vectors while the real message is that we need to make better use of the knowledge we now have in terms of designing vectors and gene therapy protocols. Clearly, the known oncogenic potential of MoMLV and its relationship to viral sequences has, for one reason or another, been largely ignored to date. Indeed, most of the retroviral vectors used in trials to date are based on MoMLV and contain an intact 5' LTR. While the historic reasons for this are obvious we now need to evaluate and adopt more appropriate technologies as rapidly as possible.

There are several obvious conclusions to be drawn from the X-SCID trial and the results of Li *et al *[71]. The first is that in the absence of additional risk factors the risk of malignant events resulting from exposure to a replication defective retroviral vector is low but remains to be accurately quantified. Secondly, what constitutes an additional risk factor is hard to predict, making risk assessment difficult. However, even with these unknowns, there exist technological approaches that should greatly reduce the risks associated with retroviral mediated gene therapy. These include SIN vectors and new types of retroviral vectors (namely lentiviral vectors) that may allow simpler transduction protocols that perturb the normal state of the target cell less than current approaches. This is especially true when the PHSC is the target of gene transfer; current protocols using oncogenic retroviral vectors rely heavily on manipulating the state of the target cell by exposure to multiple cytokines over relatively long periods. These protocols are also relatively inefficient; this reflects the poor match between the target cell and properties of these vectors. In contrast, vectors derived from lentiviruses and spumaviruses appear to allow more efficient transduction of PHSC with less requirement for cytokine stimulation of the target cells [85,86,95-97]. It is of note that the use of lentiviral vectors may also be preferable in other ways. Not only do they have uniquely positive properties as gene therapy vectors, there is no evidence that the viruses from which they are derived are able to induce gene activation using the same mechanisms as used by non-defective oncogenic retroviruses.

Regulatory authorities also have a role to play. Clinical trials are based on extensive preclinical experimentation and animal trials that take many years to complete. Clearly, the particular vector system that has been used to develop a protocol may no longer be the best to use ten years later when clinical trials become a reality. The question is, then, how can the regulation of clinical trials be made flexible enough to allow the introduction of new and improved vector technology late in the process?

In conclusion, retroviral mediated gene transfer remains an extremely attractive option for gene therapy when the stable and permanent genetic modification of the target cell is optimal. However, we must take greater care, and utilise more resources, for the pro-active, rather than reactive, refinement and testing of the basic technology that is used for gene therapy and for the adoption of improved vector systems if adverse events are to be minimised.
